# Exercise challenge alters Default Mode Network dynamics in Gulf War Illness

**DOI:** 10.1186/s12868-019-0488-6

**Published:** 2019-02-21

**Authors:** Rakib U. Rayhan, Stuart D. Washington, Richard Garner, Kristina Zajur, Florencia Martinez Addiego, John W. VanMeter, James N. Baraniuk

**Affiliations:** 1 Department of Physiology and Biophysics, Howard University College of Medicine, Adams Building Rm 2420, 520 W Street NW, Washington, DC 20059 USA; 2 Center for Functional and Molecular Imaging, Georgetown University Medical Center, 3900 Reservoir Road Suite LM14, Washington, DC 20007 USA; 3 Chronic Pain and Fatigue Research Center, Georgetown University Medical Center, Pre-Clinical Science Building, Rm LD3, 3800 Reservoir Road NW, Washington, DC 20007 USA

**Keywords:** GWI, Exercise, PEM, fMRI, Default Mode Network

## Abstract

**Background:**

Gulf War Illness (GWI) affects 30% of veterans from the 1991 Gulf War and has no known cause. Everyday symptoms include pain, fatigue, migraines, and dyscognition. A striking syndromic feature is post-exertional malaise (PEM). This is recognized as an exacerbation of everyday symptoms following a physically stressful or cognitively demanding activity. The underlying mechanism of PEM is unknown. We previously reported a novel paradigm that possibly captured evidence of PEM by utilizing fMRI scans taken before and after sub-maximal exercises. We hypothesized that A) exercise would be a sufficient physically stressful activity to induce PEM and B) Comparison of brain activity before and after exercise would provide evidence of PEM’s effect on cognition. We reported two-exercise induced GWI phenotypes with distinct changes in brain activation patterns during the completion of a 2-back working memory task (also known as two-back > zero-back).

**Results:**

Here we report unanticipated findings from the reverse contrast (zero-back > two-back), which allowed for the identification of task-related deactivation patterns. Following exercise, patients developed a significant increase in deactivation patterns within the Default Mode Network (DMN) that was not seen in controls. The DMN is comprised of regions that are consistently down regulated during external goal-directed activities and is often altered within many neurological disease states.

**Conclusions:**

Exercise-induced alterations within the DMN provides novel evidence of GWI pathophysiology. More broadly, results suggest that task-related deactivation patterns may have biomarker potential in Gulf War Illness.

**Electronic supplementary material:**

The online version of this article (10.1186/s12868-019-0488-6) contains supplementary material, which is available to authorized users.

## Background

Following the 1991 Persian Gulf War, deployed veterans began to report general symptoms of widespread pain, fatigue, migraines, dyscognition, and other interoceptive complaints [1, 2]. It is estimated that nearly one-third of deployed veterans continue to suffer from this syndrome widely described as Gulf War Illness (GWI) [3]. Commonly accepted diseases have been excluded as the etiological cause and the underlying mechanism is still unknown. Current evidence suggests deployment related toxicant exposures might be responsible for GWI symptomatology [3]. Clinically defining this syndrome has been difficult because patients present with heterogeneous combinations of symptoms [4].

A notable exception to the clinical heterogeneity is the cardinal feature known as post exertional malaise (PEM) [4, 5]. Often described as a crash, patients report that symptoms dramatically worsen following a physically demanding or cognitively challenging activity [6]. The pathophysiology underlying this stressor-induced response is unknown. Due to the intense symptom fluctuations among patients at baseline, examining GWI in this exacerbated state allows for a more homogenous clinical presentation for study [7]. Despite an unknown etiology, a multitude of functional magnetic resonance imaging (fMRI) studies (including our own) strongly suggest that alterations in brain structure and function play a prominent role in GWI pathophysiology [5, 8–10].

Neuroimaging studies have consistently reported two general responses during the completion of a task: (1) The increase (activation) of the BOLD signal in task-related regions and (2) The decrease (deactivation) of task-negative regions known as the Default Mode Network (DMN) [11, 12]. The DMN is most active during times of rest. Anatomically, it is housed within the medial prefrontal, bilateral parietal and temporal cortices [11]. Functionally, the DMN supports recollection, emotional processing, and self-referential mental activity [12]. It has become a prominent component of cognitive neuroscience due to its consistent deactivation regardless of the type of cognitive task being presented [13]. Not only is the DMN “turned-off” or suppressed, but also the magnitude of its deactivation is dependent upon task difficulty [14]. Further, alterations within the DMN have been reported in a vast array of neurological disorders, suggesting its central importance in normal cognition [12].

We previously reported findings from a novel longitudinal study that utilized fMRI brain scans before and after submaximal exercise as a viable model for PEM in 28 GWI subjects and 10 controls [5].

All participants completed a cognitively demanding two-back working memory exam before and after exercise. We employed a traditional blocked design for our working memory task with two-levels (two-back and zero-back). To isolate the BOLD activity that was significant during the two- back condition (task-related activation), we subtracted the control condition (zero-back) from the task of interest (two-back > zero-back) [5, 15].

We reported two distinct GWI subgroups with unique cardiovascular and functional brain pattern changes during the 2-back task [5]. Ten GWI subjects developed transient post-exercise postural tachycardia that lasted for 36 to 48 h with subsequent reduction in BOLD activity during the two-back task [5]. They were defined as the Stress Test Activated Reversible Tachycardia (START) phenotype. The remaining 18 GWI subjects developed post-exertional brain activation patterns consistent with phantom limb pain and were called the Stress Test Originated Phantom Perception (STOPP) phenotype. Controls did not show any clinical or brain activation differences following exercise.

In this current article, we report novel and unanticipated findings from isolating the reverse contrast (zero-back > two-back). This contrast specifically shows task-related deactivation. Prior to exercise, there was no detectable differences between the groups. However, following exercise there was a dramatic increase of task-related deactivation patterns within the DMN in GWI patients but not controls. Our novel results provide further objective evidence for the subjective feature known as PEM and strongly suggests that GWI is a central nervous system disorder.

## Results

### Demographics, phenotype Identification, and task accuracy

**Fig. 1:**
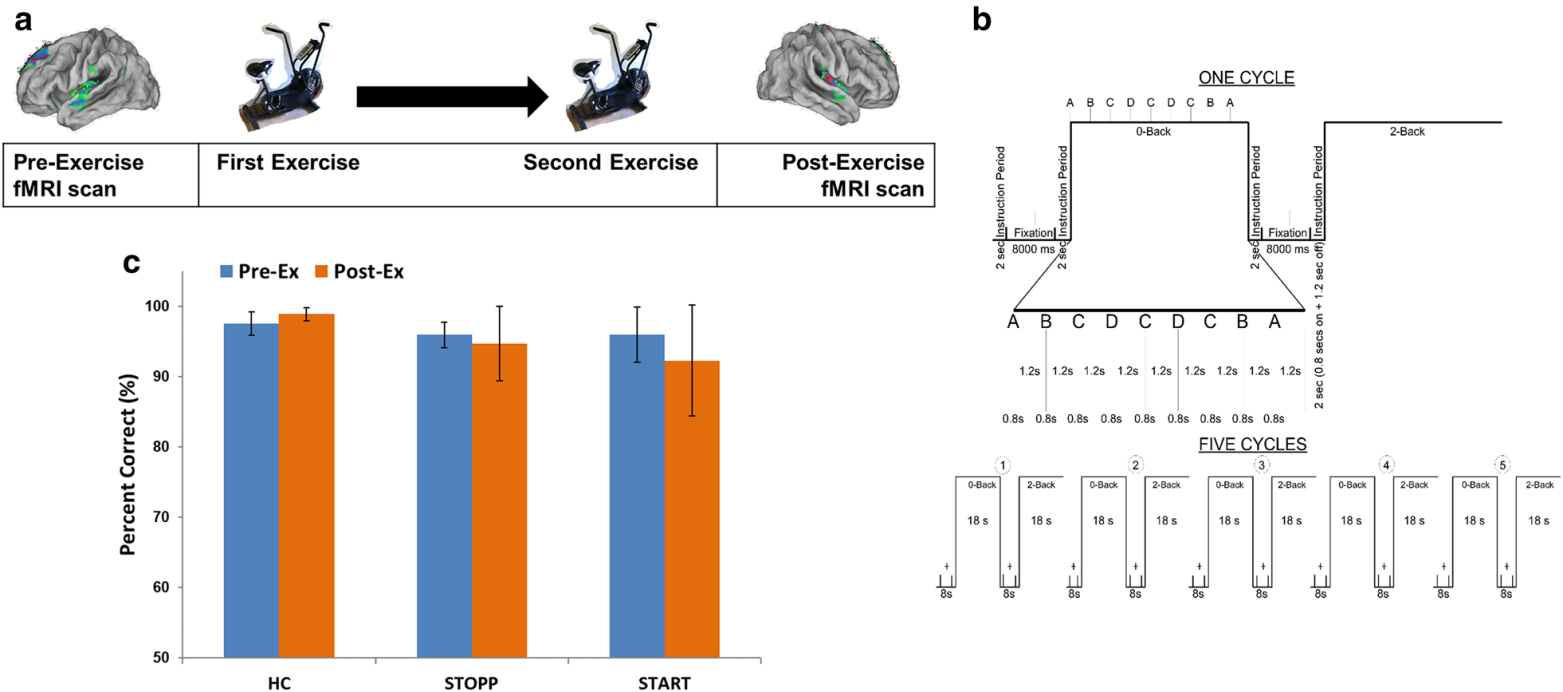
Protocol setup and accuracy. **a** Schematic of fMRI-exercise protocol. **b** Experimental N-back task design and timing intervals. **c** 0-back accuracy. There was no significant difference between HC, STOPP, or START subjects before and after exercise. Bicycle images were generated by staff of the actual bicycle used during the protocol

There were no significant differences in demographic variables between GWI (n = 28) and controls (n = 10) (Table 1). The 28 GWI subjects were subdivided into two groups following exercise [5]. Ten GWI subjects developed postural tachycardia after exercise and were labeled the START group (n = 10). The remainder formed the STOPP group (n = 18). Exercise induced changes in the experimental measures were absent in controls. There were no significant differences between groups on accuracy across both days (Fig. 1c).

**Table 1:** Demographics of participants

Groups	Controls	STOPP	START
N=	10	18	10
Age	48.9 [42.8 to 55.0]	45.8 [42.3 to 49.3]	44.4 [49.6 to 39.2]
Gender			
Male	8	13	9
Female	2	5	1

### Neuroimaging results

#### Pre-exercise 0-back activity

**Fig. 2:**
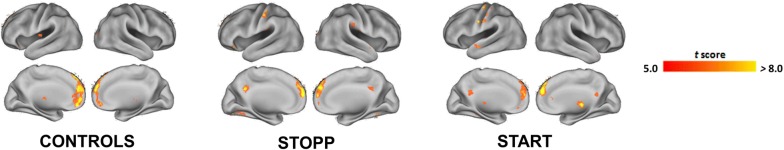
Significant deactivation during the 0-back condition (0 > 2-back contrast) before exercise. Prior to exercise controls and GWI subgroups demonstrated similar deactivation patterns within the medial prefrontal cortex (*P *< 0.05, clusterwise corrected threshold)

Within group analysis of zero-back BOLD activity (zero-back > two-back) revealed controls and GWI subgroups deactivated regions (*p* < 0.05, FDR) within the bilateral rostral-medial prefrontal cortex (mPFC) (Additional file 1: Table S1). A direct between group comparison of controls and GWI subgroups indicated similar clusters of activation (Fig. 2) within regions comprising the mPFC (Table 2).

**Table 2:** Significant clusters of deactivation before the exercise stressors

Pre-exercise fMRI			
Group	Cluster Size	T-score peak voxel	MNI Coordinates*
*Controls (n = 10)*			
Bilateral dorsomedial prefrontal cortex	769	9.07	4, 44, 52 − 10, 50, 38 − 6, 40, 56
Left ventromedial prefrontal cortex	161	6.25	− 8, 50, − 14 − 2, 54, 4 − 10, 54, − 6
*STOPP subjects (n = 18)*			
Bilateral dorsomedial prefrontal cortex	1413	6.11	− 2, 60, 12 4, 52, 44 − 6, 62, 22
START subjects (n = 10)			
Right ventromedial prefrontal cortex	323	7.57	10, 54, 42 8, 54, 28

Prior to exercise, controls and GWI groups deactivated similar regions within the medial prefrontal cortex

* Greater than 1 reported MNI coordinate shows separate (> 8 mm apart) local maxima within a cluster. (*P *< 0.05, clusterwise corrected threshold; MNI coordinates x, y and z in mm)

#### Post-exercise 0-back activity

**Fig. 3:**
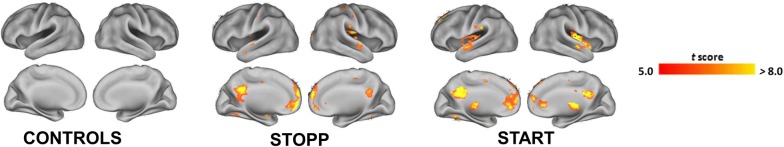
Significant deactivation during the 0-back condition (0 > 2-back contrast) after exercise. Following exercise controls did not have any significant BOLD activity. In contrast, GWI subgroups demonstrated similar and robust deactivation patterns in DMN regions such as the precuneus and medial prefrontal cortex (*P *< 0.05, clusterwise corrected threshold)

Following exercise, controls did not demonstrate significant within or between group level deactivations, possibly implying automaticity or learning of task (Fig. 3) [16]. In contrast, within group analysis (Additional file 1: Table S2) of START and STOPP subgroups (*p *< 0.05, FDR) showed widespread and significant deactivation throughout the brain (Fig. 3). A direct between group comparisons showed STOPP subjects had significant clusters of deactivation (*p *< 0.05, cluster corrected threshold) in the bilateral ventromedial prefrontal cortex (vmPFC), bilateral precuneus, and left posterior insula (Table 3). START subjects had significant clusters of deactivation (*p *< 0.05, cluster corrected threshold) in the left dorsomedial prefrontal cortex (dmPFC), left precuneus, right posterior insula, right amygdala, and right Thalamic nuclei (Table 3). Both START and STOPP groups shared prominent deactivation patterns within regions associated with the DMN (Fig. 3).

**Table 3:** Significant clusters of deactivation after the exercise stressors

Post-exercise fMRI			
STOPP subjects (n = 18)	Cluster size	T-score peak voxel	MNI Coordinates*
Bilateral ventromedial prefrontal cortex	1245	6.29	− 4, 62, 0 − 2,56,− 8 4,62,24
Bilateral precuneus	330	6.00	− 8, − 48, 28 8, − 48, 28
Left posterior insula	103	5.10	− 42, − 16,20 − 36, − 8, 16
*START subjects (n = 10)*			
Left dorsomedial prefrontal cortex	134	5.53	− 20, 50,38 − 20, 36, 44
Left precuneus	527	9.38	− 14, − 56, 28 − 10, − 48, 34 − 6, − 52, 24
Right posterior insula	271	8.97	38, − 14, 12 40, − 8, − 4 50, 0, 4
Right amygdala and right posterior insula	136	6.67	32, 8,− 16 40, 6, 12
Right MDN and Pulvinar of Thalamus	228	6.52	4, − 20, 8 14, − 26, 6

In addition, both subgroups exhibited phenotypically exclusive clusters (Table 3). Most notably, START subjects had significant findings within deep brain nuclei (thalamus and amygdala) and right posterior insula (*p *< 0.05, cluster corrected threshold). The STOPP group had significant deactivation within the left posterior insula (*p *< 0.05, cluster corrected threshold). Results suggest that physically demanding stressors can profoundly alter brain networks in GWI subjects even during a simple stimulus-matching task such as the zero- back control condition.

Following exercise, controls did not show any significant deactivation clusters. GWI subgroups shared extensive deactivation in DMN associated regions and also had phenotypic specific regions of deactivation. *Greater than 1 reported MNI coordinate shows separate (> 8 mm apart) local maxima within a cluster. (*p *< 0.05, cluster corrected threshold; MNI coordinates x, y and z in mm).

## Discussion

We sought to model PEM in GWI and its impact on brain function using our novel fMRI- exercise protocol. Our results show that physical exertion leads to widespread changes in activity and provides neural surrogates for the symptom of PEM in GWI. Data also indicates that controls did not experience similar changes in cognition following exercise. Prior to exercise, task-related deactivation in the rostral medial PFC was not significantly different between groups. This is noteworthy, as cross-sectional fMRI studies without a physical stressor may not be adequate for differentiating GWI from the general population.

Following exercise, prominent task-related DMN deactivation was not detectable in controls. This suggests the development of automaticity and learning [16, 17]. In contrast, both GWI subgroups had significant task-related deactivations within the mPFC and precuneus. Both are prominent regions within the DMN [11, 13]. This may reflect the lack of automaticity as GWI subgroups did not learn the task as well as controls [18–21]. However, findings may implicate the opposing situation: greater automaticity during the 0-back. The increased DMN activity may in fact be GWI subjects partaking in mind-wandering activities as they found the 0-back task to be not difficult [22]. Current data showing exercise-induced changes in DMN activity coupled with previous reports of altered working memory activity [5] suggests a physiological stressor has definable effects on large-scale neural networks in GWI.

Increases in the magnitude of deactivation within the DMN is normally associated with increases in task difficulty [23, 24]. Our GWI subjects developed higher levels of DMN deactivation following exercise despite completing the same task [5]. In addition, GWI subgroups did not significantly differ in their accuracy (Fig. 1a). To reconcile the finding of post-exertional elevation in task-related DMN deactivation with no change in accuracy, GWI subjects may have had to increase their effort to maintain the same level of cognitive work [25]. Clinically, GWI patients describe PEM as a consistent fog that clouds their abilities do to cognitive tasks [4–6]. Post-exertional increase in the task-related deactivation of the DMN may provide objective evidence for this subjective complaint in GWI. Decreases in accuracy with the increases in task-related DMN deactivation may be seen for tasks that are more difficult and future studies should explore this possibility.

Two of the most prominent symptoms that are sensitive to physiological stressors in GWI are fatigue and pain [2–5]. Previous reports show that persistent fatigue and mental exhaustion are positively correlated with DMN functional connectivity [26, 27]. Both STOPP and START subgroups also showed task-related deactivation within non-DMN nodes such as the posterior insula, which is implicated in processing pain salient stimuli [28]. START subjects also showed task-related deactivation in deep-brain nuclei, which is active during prolonged attention towards a task [29]. Therefore, it is strongly plausible that the increase in task-related deactivation in GWI patients may represent the attempt to suppress the distracting effects of PEM in order to reallocate resources and complete the external goal- directed task [30].

We did not complete a true resting state scan and are unable to state whether the DMN was altered at baseline. In addition, the fMRI BOLD patterns only provide evidence for what is activated or deactivated but not the correlational relationships between brain regions. Our small sample size is a limitation for this initial analysis. Future studies will need to replicate findings in a larger sample and incorporate a functional resting state scan into the protocol.

The association between post-exertional increases in task-related DMN deactivation may have important implications for GWI and other fatiguing illnesses where evidence of disease pathology have been difficult to elucidate [4, 6]. Thus, our findings may have broad appeal.

## Conclusion

Exercise induced increases in the task-induced deactivation of the DMN support our previous reports and may serve as a novel biomarker for the previously ill- defined symptom of PEM in GWI.

## Methods

### Subjects

Ten healthy sedentary veterans plus civilian controls (HC) and 28 GWI subjects gave written informed consent to complete the protocol that was approved by the Georgetown University Institutional Review Board and Department of Defense Human Research Protection Office. The prior published paper from our group, which incorporated the same 10 HC and 28 GWI subjects used in this report, includes extensive details of the protocol, demographics and other related information [5].

### Experimental Design

Subjects completed two bicycle exercise stress tests on consecutive days as previously reported [5]. Brain scans were acquired before and after the two stress tests. A schematic summarizes the 4-day protocol (Fig. 1a). During fMRI acquisition, structural and functional BOLD data were obtained while participants completed the N-back working memory task.

### MR scanning acquisition

Pre-exercise and post-exercise scans were collected on a Siemens 3-Tesla Tim Trio scanner with a standard 12-channel head coil array. During the N-back task the blood oxygenation level dependent (BOLD) signal was attained using a T2*-weighted gradient-echo planar imaging (EPI) with the following imaging parameters: TE/TR = 30 ms/2500 ms, 90° flip angle, 64 × 64 acquisition matrix, field of view (FOV) = 205 mm^2^, voxel resolution = 3.2 mm^3^ and 47 slices.

High-resolution T1-weighted anatomical scans were acquired with a three-dimensional magnetization prepared rapid acquisition gradient echo (MPRAGE) sequence with the following imaging parameters: TE/TR = 2.52 ms/1900 ms, TI  =  900 ms, FOV  =  250 mm^2^, slice resolution = 1.0 mm^3^, and 176 slices.

### N-back experimental paradigm

Participants completed an N-back working memory task with two levels before and after exercise (Fig. 1b): a two-back and zero-back portion [5, 15]. The fMRI results from the two-back portion were already published [5]. This current report specifically focuses on the fMRI activity from the zero-back portion.

Alternating blocks, presented in 5 cycles, of zero-back and two-back tasks were presented to participants before and after exercise. At the start and between each zero-back and two-back blocks, participants viewed a blank screen with a screen-centered cross-hair projection that spanned 8000 ms (with no task presentation). This was followed by a brief display of on-screen instructions. Within each block, nine pseudo-randomized letters (A, B, C, and D) were presented for 1000 ms followed by 1500 ms of a blank screen (Fig. 1b). In the zero-back stimulus-matching portion of the task, subjects were asked to press the button that corresponds to the letter currently displayed. During the two-back working memory task, subjects were instructed to press the button for two letters previously [5, 15]. The N-back task was presented using the E-prime software package (Psychology Software Tools, Inc). A projector and a mirror attached to the head coil was used to view the N-back paradigm.

### fMRI preprocessing

SPM5 (http://www.fil.ion.ucl.ac.uk/spm/software/spm5/) in MATLAB (R2016b) was used for preprocessing and statistical analysis. First, raw DICOM images were converted to the nifti format. Second, preprocessing entailed correction for Siemens’ interleaved slice timing sequence. Then, motion correction entailed spatial realignment of all images (source images) to the first fMRI volume (reference image) using a six-parameter rigid-body transformation (three translations (mm) and three rotations (degrees)). Realigned within-subject images were next co-registered to their respective high-resolution T1-weighted MPRAGE anatomical image. The anatomical MPRAGE image was then segmented and subsequently transformed into the MNI standard stereotactic space using linear regularization. Parameters from the linear regularization were applied to normalize fMRI images into the MNI space. Data was spatially smoothed using a Gaussian kernel of 5 mm^3^ full-width half maximum (FWHM). Subjects were excluded from analysis if 20% of their total volumes met the definition for a motion artifact. We defined motion artifacts as any volume with translational or rotational movement that was greater than two standard deviations from the mean.

### First-level fMRI analysis

In SPM5, we used a previously described masking procedure to identify regions of neural activity, thresholded at *P *< 0.001 uncorrected, for each component of the paradigm (zero-back and two-back) [5]. The contrast of interest was zero-back greater than two-back (0-back > 2-back). The newly created masks were then entered as regressors into each subject’s first level design matrix. For these analyses, we also included each subject’s six-motion parameters as nuisance regressors for movement. Finally, the contrast of 0-back > 2-back for each participant was carried out to highlight regions of activity specific to the 0-back portion of the paradigm.

### Second-level (group) fMRI analysis

To identify significant regions (corrected for multiple comparisons) of BOLD activity between groups and across days, we used the AFNI based 3dClustSim program (AFNI 16.3.03). The version used in this analysis includes updates in response to Eklund et al. publication of possible bugs within prior versions [31]. Using 3dClustSim, we conducted 10,000 Monte Carlo simulations. For a given whole-brain search space and voxel-wise probability threshold, this method provides the needed cluster volume to retain the desired false-positive rate for cluster detection. Using a voxel-wise threshold of *P* < 0.001 uncorrected and smoothness with a FWHM of 13 mm^3^, a cluster volume threshold of 90 contiguous voxels was significant to hold the probability of map-wise false-positive detection at *P* < 0.05 in the whole-brain analyses. For significant clusters, we reported MNI coordinates that corresponded to the peak voxel t-score. Group activation maps were generated and displayed onto the standard caret brain (citation) with corresponding t-value scaling as previously reported [5]. Means were calculated and are reported as [± 95% confidence intervals (C.I.)].

## Additional file

**Additional file 1.** Significant voxel-wise regions before/after exercise. Significant voxels per group and across days organized within tables. Includes corresponding anatomical regions and MNI coordinates.

## Abbreviations

BOLD: blood oxygen level dependent
DMN: Default Mode Network
FDR: false discovery rate
fMRI: functional magnetic resonance imaging
GWI: Gulf War Illness
PEM: post-exertional malaise
START: stress test activated reversible tachycardia
STOPP: stress test originated phantom perception
