# Supplementary material for: Exercise challenge alters Default Mode Network dynamics in Gulf War Illness
Source: BMC Neurosci. 2019 Feb 21;20:7. doi: 10.1186/s12868-019-0488-6 (PMC6385399; doi:10.1186/s12868-019-0488-6)
Supplement: Supplementary file 1 — Additional file 1. Significant voxel-wise regions before/after exercise. Significant voxels per group and across days organized within tables. Includes corresponding anatomical regions and MNI coordinates. [file 12868_2019_488_MOESM1_ESM.docx]

Supplementary Material

**Supplementary Table 1 (Table S1). Coordinates of significant voxel-wise regions during completion of the 0-back paradigm before exercise.**

|  |  | Control (N=10) | | | | | STOPP (N=18) | | | | | START (N=10) | | | | |
| --- | --- | --- | --- | --- | --- | --- | --- | --- | --- | --- | --- | --- | --- | --- | --- | --- |
| Hemisphere | Location | T | BA | x | y | z | T | BA | x | y | z | T | BA | x | y | z |
| Left Cerebrum | Superior frontal gyrus | 8.24 | 8 | -10 | 50 | 38 | 5.81 | 9 | -5 | 52 | 22 | 5.10 | 9 | -10 | 50 | 20 |
|  | Medial frontal gyrus | 6.27 | 10 | -8 | 50 | -14 | 6.11 | 10 | -2 | 60 | 12 | 5.81 | 10 | -14 | 48 | 6 |
|  | Inferior frontal gyrus |  |  |  |  |  | 4.73 | 47 | -40 | 30 | -14 |  |  |  |  |  |
|  | Dorsal Ant Cingulate | 4.82 | 32 | -16 | 48 | -4 |  |  |  |  |  |  |  |  |  |  |
|  | Dorsal Post cingulate |  |  |  |  |  |  |  |  |  |  | 4.35 | 31 | -10 | -44 | 26 |
|  | Pre-central gyrus |  |  |  |  |  |  |  |  |  |  | 4.81 | 6 | -40 | -14 | 62 |
|  | Post-central gyrus |  |  |  |  |  | 4.64 | 2 | -48 | -28 | 56 |  |  |  |  |  |
|  | Insula, Posterior |  |  |  |  |  | 4.64 | 13 | -48 | -22 | 20 |  |  |  |  |  |
|  | Precuneus |  |  |  |  |  | 5.27 | 31 | -10 | -52 | 28 |  |  |  |  |  |
|  | Fusiform gyrus |  |  |  |  |  | 5.19 | 19 | -30 | -76 | -12 | 4.46 | 37 | -34 | -38 | -14 |
|  | Inferior Parietal Lobule |  |  |  |  |  | 3.79 | 40 | -55 | -28 | 22 |  |  |  |  |  |
|  |  |  |  |  |  |  |  |  |  |  |  |  |  |  |  |  |
|  |  |  |  |  |  |  |  |  |  |  |  |  |  |  |  |  |
|  |  |  |  |  |  |  |  |  |  |  |  |  |  |  |  |  |
| Right Cerebrum | Superior frontal gyrus | 9.07 | 8 | 4 | 44 | 54 |  |  |  |  |  | 7.57 | 9 | 10 | 54 | 42 |
|  | Medial frontal gyrus |  |  |  |  |  | 5.81 | 8 | 4 | 52 | 44 |  |  |  |  |  |
|  | Post-central gyrus |  |  |  |  |  | 3.87 | 43 | 56 | -14 | 20 |  |  |  |  |  |
|  | Inferior Parietal Lobule |  |  |  |  |  | 4.40 | 40 | 62 | -28 | 30 |  |  |  |  |  |
|  | Middle occipital gyrus | 7.06 | 19 | 36 | -88 | 14 |  |  |  |  |  |  |  |  |  |  |
|  |  |  |  |  |  |  |  |  |  |  |  |  |  |  |  |  |
|  |  |  |  |  |  |  |  |  |  |  |  |  |  |  |  |  |
|  |  |  |  |  |  |  |  |  |  |  |  |  |  |  |  |  |
|  |  |  |  |  |  |  |  |  |  |  |  |  |  |  |  |  |
|  |  |  |  |  |  |  |  |  |  |  |  |  |  |  |  |  |
| Left Cerebellum | Inferior semi-lunar lobule | 4.67 |  | -24 | -82 | -38 | 4.39 |  | -24 | -82 | -38 |  |  |  |  |  |
|  |  |  |  |  |  |  |  |  |  |  |  |  |  |  |  |  |
| Right Cerebellum | declive |  |  |  |  |  | 4.16 |  | 26 | -52 | -14 |  |  |  |  |  |

Controls and GWI subgroups activated regions withinin the medial prefrontal cortex. (T=T value; BA=Brodmann's area; MNI coordinates x, y and z in mm). Whole-brain maps were voxel corrected at *p*<0.05, False discovery rate; FDR).

**Supplementary Table 2 (Table S2). Coordinates of significant voxel-wise regions during completion of the 0-back paradigm after exercise.**

|  |  | Control (N=10) | | | | | STOPP (N=18) | | | | | START (N=10) | | | | |
| --- | --- | --- | --- | --- | --- | --- | --- | --- | --- | --- | --- | --- | --- | --- | --- | --- |
| Hemisphere | Location | T | BA | x | y | z | T | BA | x | y | z | T | BA | x | y | z |
| Left Cerebrum | Superior frontal gyrus |  |  |  |  |  | 6.29 | 8 | -4 | 62 | 0 | 5.53 | 8 | -20 | 50 | 38 |
|  | Middle frontal gyrus |  |  |  |  |  | 5.54 | 11 | -34 | 38 | -10 |  |  |  |  |  |
|  | Medial frontal gyrus |  |  |  |  |  | 6.24 | 10 | -2 | 56 | -8 | 4.65 | 10 | -8 | 54 | 6 |
|  | Dorsal Ant Cingulate |  |  |  |  |  |  |  |  |  |  | 4.76 | 32 | -8 | 44 | -4 |
|  | Insula, Posterior |  |  |  |  |  | 4.67 | 13 | -38 | -8 | 15 | 5.00 | 13 | -40 | -2 | 12 |
|  | Dorsal Post Cingulate |  |  |  |  |  | 3.38 | 29 | -6 | -54 | 12 | 6.92 | 31 | -5 | -52 | 24 |
|  | Post-central Gyrus |  |  |  |  |  | 3.83 | 43 | -50 | -25 | 22 | 5.57 | 43 | -52 | -14 | 18 |
|  | Precuneus |  |  |  |  |  | 6.00 | 31 | -8 | -48 | 28 | 9.38 | 31 | -14 | -56 | 28 |
|  | Cuneus |  |  |  |  |  | 4.62 | 19 | -10 | -92 | 30 |  |  |  |  |  |
|  | Inferior Parietal Lobule |  |  |  |  |  |  |  |  |  |  | 6.39 | 40 | -50 | -30 | 24 |
|  | Para-central lobule |  |  |  |  |  | 4.06 | 31 | -4 | -12 | 44 |  |  |  |  |  |
|  | Amygdala |  |  |  |  |  | 4.74 |  | -24 | -4 | 14 |  |  |  |  |  |
|  | Parahippocampal gyrus |  |  |  |  |  | 4.28 | 37 | -28 | -44 | -8 | 7.78 | 34 | -24 | 0 | -12 |
|  | Claustrum |  |  |  |  |  |  |  |  |  |  | 5.66 |  | -38 | -16 | -4 |
| Right Cerebrum | Superior frontal gyrus |  |  |  |  |  |  |  |  |  |  | 7.13 | 8 | 10 | 46 | 52 |
|  | Middle frontal gyrus |  |  |  |  |  | 4.90 | 47 | 40 | 36 | -10 | 4.94 | 11 | 34 | 34 | -10 |
|  | Medial frontal gyrus |  |  |  |  |  | 5.55 | 9 | 4 | 52 | 24 | 4.60 | 10 | 8 | 54 | 12 |
|  | Inferior frontal Gyrus |  |  |  |  |  |  |  |  |  |  | 4.86 | 47 | 25 | 30 | -10 |
|  | Ventral Ant Cingulate |  |  |  |  |  |  |  |  |  |  | 5.08 | 24 | 4 | 36 | -2 |
|  | Insula, Posterior |  |  |  |  |  | 5.10 | 13 | 42 | -16 | 20 | 8.97 | 13 | 38 | -14 | 12 |
|  | Post-central Gyrus |  |  |  |  |  | 4.46 | 40 | 56 | -24 | 24 |  |  |  |  |  |
|  | Precuneus |  |  |  |  |  | 4.19 | 21 | 8 | -48 | 28 |  |  |  |  |  |
|  | Cuneus |  |  |  |  |  | 4.71 | 18 | 16 | -96 | 18 |  |  |  |  |  |
|  | Superior Temp Gyrus |  |  |  |  |  | 3.79 | 41 | 44 | -30 | 15 | 6.66 | 38 | 50 | 0 | 4 |
|  | Middle Temp Gyrus |  |  |  |  |  | 3.88 | 21 | 50 | -8 | -14 |  |  |  |  |  |
|  | Transverse Temp Gyrus |  |  |  |  |  |  |  |  |  |  | 6.31 | 42 | 60 | -12 | 16 |
|  | Middle Occipital Gyrus |  |  |  |  |  | 4.89 | 19 | 30 | -92 | 16 |  |  |  |  |  |
|  | Amygdala |  |  |  |  |  | 4.18 |  | 30 | -5 | -10 |  |  |  |  |  |
|  | Parahippocampal gyrus |  |  |  |  |  | 4.65 | 34 | 28 | 2 | -10 |  |  |  |  |  |
|  | Thalamus: MDN |  |  |  |  |  |  |  |  |  |  | 6.52 |  | 4 | -20 | 8 |
|  | Thalamus: Pulvinar |  |  |  |  |  |  |  |  |  |  | 5.21 |  | 14 | -25 | 5 |
| Left Cerebellum | Culmen |  |  |  |  |  |  |  |  |  |  | 6.43 |  | -32 | -52 | -16 |
| Right Cerebellum | Pyramis |  |  |  |  |  | 4.16 |  | 32 | -78 | -34 |  |  |  |  |  |

Controls did not show any voxel wise activation following exercise. In contrast, GWI subgroups activated regions larger regions within the medial prefrontal cortex and precuneus. MDN=Medial Dorsal Nucleus; (T=T value; BA=Brodmann's area; MNI coordinates x, y and z in mm). Whole-brain maps were voxel corrected at *P*<0.05, False discovery rate; FDR).
